# Stress exposure alters brain mRNA expression of the genes involved in insulin signalling, an effect modified by a high fat/high fructose diet and cinnamon supplement

**DOI:** 10.1371/journal.pone.0197094

**Published:** 2018-05-29

**Authors:** Frédéric Canini, Bolin Qin, Nathalie Arvy, Laurent Poulet, Cécile Batandier, Anne-Marie Roussel, Richard A. Anderson

**Affiliations:** 1 Département Neurosciences & Contraintes Opérationnelles, Institut de Recherche Biomédicale des Armées (IRBA), Brétigny-sur-Orge Cedex, France; 2 École du Val de Grâce, 1 place Laveran, Paris, France; 3 In-ingredients.com, Columbia, TN, United States of America; 4 Diet, Genomics, and Immunology Laboratory, Beltsville Human Nutrition Research Center, Agricultural Research Service, United States Department of Agriculture, Beltsville, MD, United States of America; 5 INRA, Laboratory of Nutrition and Integrative Neurobiology, UMR 1286, Bordeaux Cedex, France; 6 University of Bordeaux, Laboratory of Nutrition and Integrative Neurobiology, UMR 1286, Bordeaux Cedex, France; 7 LBFA/INSERM1055, Grenoble Alpes University, Grenoble, France; Technion Israel Institute of Technology, ISRAEL

## Abstract

In occidental societies, high fat and high sugar diets often coincide with episodes of stress. The association is likely to modify brain energy control. Brain insulin signalling is rarely studied in stressed individuals consuming high fat diets. Furthermore the effects of cinnamon supplement are not known in these conditions. Therefore, we exposed rats, over a 12-week period, to a control (C) or a high fat/high fructose (HF/HFr) diet that induces peripheral insulin resistance. A cinnamon supplement (C+CN and HF/HFr +CN) was added or not. After diet exposure, one group of rats was exposed to a 30-min restraint followed by a 10-min open-field test, their combination featuring a moderate stressor, the other rats staying unstressed in their home cages. The insulin signalling in hippocampus and frontal cortex was studied through the mRNA expression of the following genes: insulin receptor (*Ir*), insulin receptor substrate (*Irs1*), glucose transporters (*Glut1* and *Glut3*), glycogen synthase (*Gys1*) and their modulators, *Akt1* and *Pten*. In C rats, stress enhanced the expression of *Ir*, *Irs1*, *Glut1*, *Gys1* and *Akt1* mRNA. In C+CN rats, stress induced an increase in *Pten* but a decrease in *Gys1* mRNA expression. In HF/HFr rats, stress was associated with an increase in *Pten* mRNA expression. In HF/HFr+CN rats, stress increased *Pten* mRNA expression but also decreased *Gys1* mRNA expression. This suggests that a single moderate stress favours energy refilling mechanisms, an effect blunted by a previous HF/HFr diet and cinnamon supplement.

## Introduction

People in western societies are chronically exposed to high fat/high sugar diets and repeatedly have to cope with life events that are moderate stressors. In rodents, chronic exposure to a high fat/high sugar diet induces peripheral insulin resistance [1] and changes brain insulin signalling [2–5]. However, the decrease in brain insulin sensitivity occurs independently from that of the periphery [6] and from adiposity [7], suggesting that a mechanism specific for the cerebral tissue may exist. When present, the brain insulin resistance is characterized by functional disturbances [3, 8] and structural abnormalities [4]. The relation between stress and brain insulin resistance is usually studied in animal models of depression and diabetes [9]. However, little is known in non pathological models. Prenatally stressed animals displaying depression-like behaviours in adulthood do not exhibit major alterations in brain insulin sensitivity [10]. Naive adult mice exposed repeatedly to foot shocks, a model of chronic high-level stress, exhibit insulin resistance in the periphery but not in the brain [11]. Repeated exposure to a moderate stress such as restraint is also followed by a peripheral insulin resistance [12]. However, the effects of single exposure to a moderate stressor on brain insulin signalling are not known.

Furthermore, the effects of a previous diet on stress-induced biological reactions have not been investigated. Evidence in humans and animals suggests that a high fat/high sugar diet and stress are interacting. Chronic stress exposure favours the emergence of both peripheral insulin resistance [13] and depression [14]. Furthermore, both are epidemiologically related [15]: patients with type 2 diabetes have a higher risk of depression than non diabetic subjects [16–19], while patients with depression also have an increased risk of diabetes [20–22], insulin resistance [23] and metabolic syndrome [24] compared to non depressed subjects. Brain inflammation and free-radical accumulation may be the crossroad for mechanisms as a high fat diet and stressor exposure enhance brain inflammation [25, 26] and free-radical levels [27–31]. In turn, free-radical accumulation favours the decrease in insulin sensitivity [32, 33], therefore triggering a vicious circle. Such evolution can be limited by polyphenols that counteract free radical consequences. For instance, cinnamon, a spice that is high in polyphenols, improves insulin sensitivity [34], alleviates peripheral insulin resistance [35], limits the stress-induced oxidation, especially in diabetic rats [36], and is neuroprotective of the cerebral tissue of rats exposed to high fat diet [5].

We hypothesized that a single moderate stress exposure would modify the brain mRNA expression of genes that are involved in insulin signalling in order to facilitate the energy supply. We also hypothesized that a previous high fat/high fructose diet would blunt this adaptive response. Lastly, we hypothesized that a cinnamon supplement would either limit the deleterious effects of a previous high fat/high fructose diet, or reduce the stress-induced adaptive responses. We designed the experiments accordingly: (i) to determine the effects of a single moderate stressor exposure on brain mRNA expression of genes involved in insulin sensitivity (ii) to evaluate the impact of a previous high fat/high sugar diet on mRNA reactions and (iii) to evaluate the effect of cinnamon on the brain mRNA expression in both normal chow and high fat/high sugar nourished rats.

## Materials and methods

### Animals

The 120 Wistar male rats (Charles River, L'Arbresle, France) were 5 weeks-old and weighed 64±1 g upon arrival at the laboratory. They were kept in a temperature-controlled room (ambient temperature: 22±1°C, relative humidity: 40–60%, 12 h light/12 h dark cycle with light at 8:00). All experimental procedures were reviewed and approved by the Institutional Ethic Committee for Animal Care (Comité d'Ethique du CRSSA, Protocol N°2008/02.1). Rats were maintained and handled in agreement with the Guide for the Care and Use of Laboratory Rats (NIH, 1985).

### Diets

The diets were purchased from SAFE (Augis, France). The control diet contained 5% cellulose, 20% casein, 25% corn starch, 25% potato starch, 16% maltodextrin, 4% soybean oil, 3.5% AIN mineral mix, 1% AIN vitamin mix, 0.3% dl methionine and 0.2% choline bitartrate. The high fat/high fructose diet was similar except the corn starch, potato starch and maltodextrin were replaced by 46% fructose and 20% lard.

The cinnamon (CN) powder (*Cinnamomum burmannii*) was purchased from McCormick Spice Co. (Baltimore, MD). The water extract of the CN contained more than 5% type A polyphenols with a tetramer, cassiatannin A, with a molecular weight of 1152 and two identified trimers, cinnamtannin B-1 and cinnamtannin D-1, with a molecular weight of 864 plus two unidentified type A trimers with a molecular weight of 864 [37–39]. The amount of CN used was based upon our previous study showing a definite effect of 20 g of CN/kg of diet in spontaneously hypertensive rats [40].

### Experimental design

**Fig 1:**
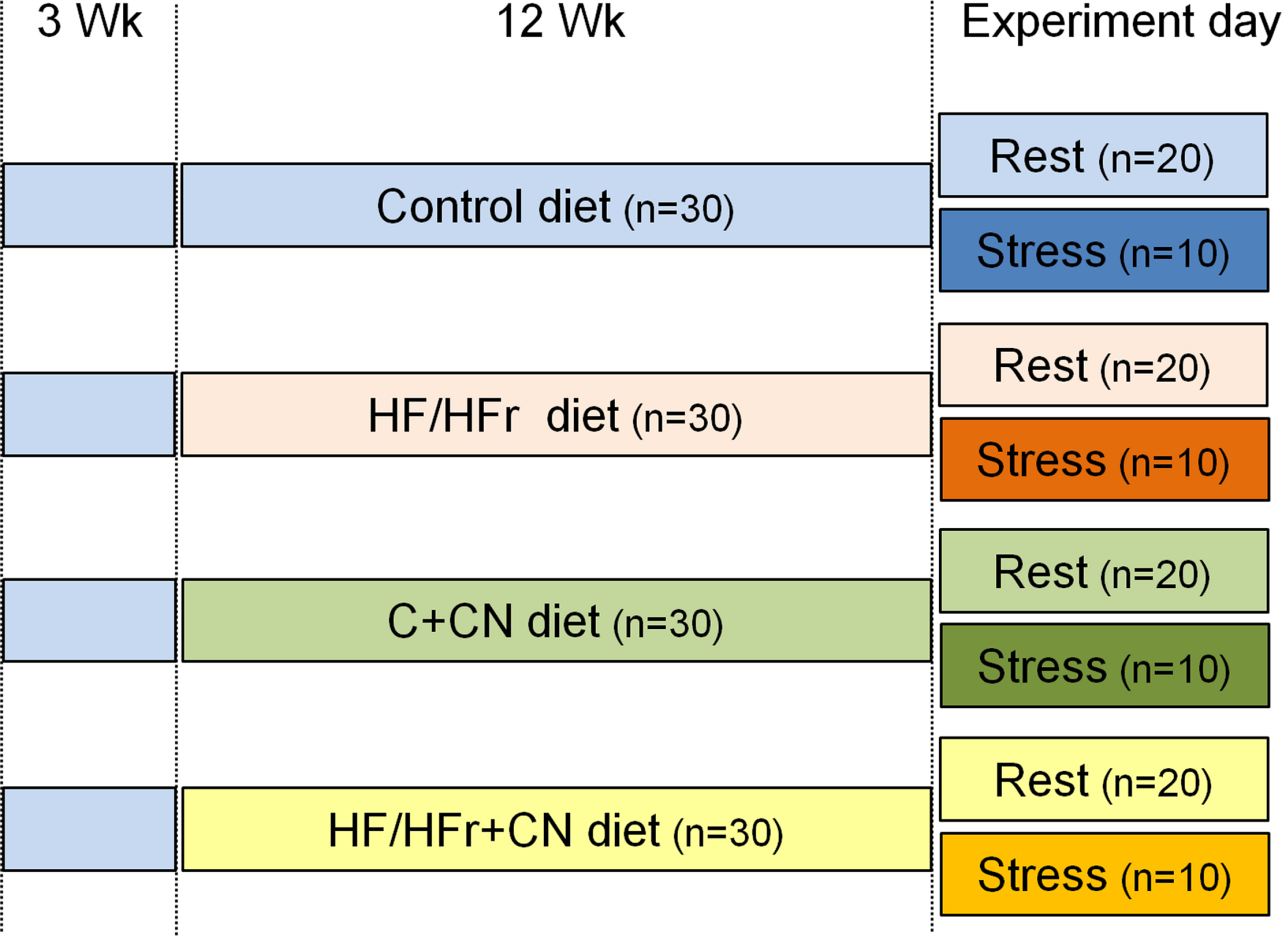
Experimental design. Diets were the following: control (C), high fat/high fructose (HF/HFr), control+cinnamon (C+CN) and high fat/high fructose+cinnamon (HF/HFr+CN). The group sizes are indicated in brackets.

After their arrival in the laboratory, the rats were familiarized with their housing environment (Fig 1). They were kept 3 per cage (26 x 40 x 15 cm) and fed a control diet for three weeks. They were then randomly divided into 4 groups and for 12 weeks they were fed *ad libitum* with one of the following four diets: control diet (C, n = 30), high fat/high fructose diet (HF/HFr, n = 30), or the respective diets containing 20 g cinnamon/kg of diet (C+CN and HF/HFr+CN, n = 30 each). During this time, they were housed individually in order to monitor their water and diet intakes. At the end of this phase, the rats from each diet group were randomly distributed into 2 sub-groups according to their exposure to stressor (Stress, n = 10) or not (Rest, n = 20). The Stress rats were submitted to a 30-min restraint immediately followed by a 10-min open field test [41]. This combination was assumed to mimic a life event stressor with the succession of movement limitation and novelty exposure. The unstressed Rest rats remained quiet in their housing cages. Stressed rats were sacrificed 1 hour following the beginning of the stress exposure. All investigations were carried out in the morning to take circadian rhythms into account.

### Acute stressor exposure

The acute stressor used was the combination of a 30-min restraint followed by an open-field test. The Rest rats were neither handled nor exposed to the combined stressor in order to keep them free from stress.

The 30-min restraint is widely used as a moderate stressor [42]. To achieve it, the rats were placed gently into a net tube and their muzzle was left free. To ensure complete restraint without any body compression, the diameter of the net was adjusted to the rat's body size. The restraint was carried out in a separate room to avoid any interaction with Rest rats.

The open-field test consisted of a white plate (100x100 cm) surrounded by a 45-cm high wall. This test was performed under a 50-lux illumination in the centre of the test. The centre of the open-field was defined as the square 15-cm away from the wall. Behaviour assessment was carried out using video tracking (Viewpoint, Lyon). Just after restraint, the animals were transferred in their home cage to a room devoted to open field test. The animal was placed in the centre of the apparatus and left free for 10 min. Before and after each test, the open-field was cleaned with 70% isopropylic alcohol. The behavioural variables used to evaluate the effect of diets on stress-induced reactivity were the following: (i) The defecation level during restraint and open-field; (ii) the locomotion in the open field as evaluated by the distance travelled and the duration of immobility periods; (iii) the propensity to go to the centre of the open field. Reduced time spent and distance travelled in the central area, long-lasting immobility in the open field and high defecation scores are indicative of anxiety [43, 44]. Locomotion speed under 5.7 cm/s was considered as immobility.

### Sacrifice and tissue sampling

After overnight fasting, the animals were transferred in their home cage to a particular room to avoid any added stress. They were quickly anesthetized with a mixture of isoflurane (3% in 100% O_2_). Blood samples were taken by cardiac puncture in a tube coated with EDTA. The brain was quickly removed and dissected on an ice bed using a glass tool. The entire hippocampus and a sample of the frontal cortex were taken and immediately frozen in liquid nitrogen and stored at -80°C until analyses.

### Biochemical determination

The concentrations of plasma corticosterone were measured with an in-house RIA using a highly specific antibody provided by H. Vaudry (University of Rouen, France) as described [45].

### mRNA determinations

The gene expression both in the hippocampus and frontal cortex were evaluated in rats randomly taken from each experimental group: C (Rest: n = 8 and Stress: n = 8), C+CN (Rest: n = 9 and Stress: n = 7), HF/HFr (Rest: n = 8 and Stress: n = 8), HF/HFr+CN (Rest: n = 8 and Stress: n = 8). The assessed mRNA expressions were the following: insulin receptor (*Ir*), [46], insulin receptor substrate (*Irs*) type I and II [46], glucose transporter type 1 (*Glut1*) and 3 (*Glut3*) [47], glycogen synthase type 1 (*Gys1*) [48] and glycogen synthase kinase (*Gsk3β*), *Akt1* protein (*Akt1*), phosphatase and tensin homolog (*Pten*), apoprotein E (*ApoE*), sterol regulatory element binding protein (*Srebp1*), tau and precursor amyloid protein (*App*).

Total RNA was isolated from hippocampus and cortex using Trizol reagent (Invitrogen, Carlsbad, CA). RNA concentrations and integrity were determined using RNA 6000 Nano Assay Kit and the Bioanalyzer 2100 according to the manufacturer’s instructions (Agilent Technologies, Santa Clara, CA). The primers used for PCR were as follows: *Ir* primers, 5’-CAAAAGCACAATCAGAGTGAGTATGAC-3’ and 5’-ACCACGTTGTGCAGGTAATCC-3’; *Irs1* primers, 5’-GCCTGGAGTATTATGAGAACGAGAA-3’ and 5’-GGGGATCGAGCGTTTGG-3’; *Irs2* primers, 5’-AAGATAGCGGGTACATGCGAAT-3’ and 5’-GCAGCTTAGGGTCTGGGTTCT-3’; *Glut1-45kDa* primers, 5’-GTGCTTATGGGTTTCTCCAAA-3’ and 5’-GACACCTCCCCCACATACATG-3’; *Glut3* primers, 5’-TTTGCAGTAGGCGGAATGG-3’ and 5’-GCCAACATGGCTTTGATCCTT-3’; *Glut3* primers: 5′-TGGCTACAACACCGGAGTCATCAA-3′ and 5′-CTGCCAAAGCGGTTGACAAAGAGT-3′; *Gys1* primers, 5’- TCCACTGTGCCTGTGTCTTCA-3’ and 5’-AGAGAACTTCTTCACATTCAGTCCATT-3’; *Gsk3β* primers, 5’-TTAAGGAAGGAAAAGGTGAATCGA-3’ and 5’-CCAAAAGCTGAAGGCTGCTG-3’; *18S* primers, 5’-TAAGTCCCTGCCCTTTGTACACA-3' and 5’-ATCCGAGGGCCTCACTAAAC-3’; *Akt1* primers: 5’-GTGGCAAGATGTGTATGAG-3’and 5’-CTGGCTGAGTAGGAGAAC-3’; *Pten* primers: 5’-ACACCGCCAAATTTAACTGC-3’ and 5’-TACACCAGTCCGTCCCTTTC-3’; *ApoE* primers: 5′-TTGGTCCCATTGCTGACAG-3′ and 5′-ACCGTCAGTTCCTGTGTGAC-3’; *Srebp1* primers: 5’-ACAAGATTGTGGAGCTCAAG-3’and TGCGCAAGACAGCAGATTTA-3’; *Tau* primers: 5′-CGGCGTAAGCAAAGACA-3′; and 5′-TGTAGCCGCTTCGTTCT-3′; *App* primers: 5’-GGATGCGGAGTTCGGACATG-3’, and 5’-GTTCTGCATCTGCTCAAAG-3’.

The mRNA levels were assessed using real-time quantitative RT-PCR. All PCR reactions were performed in a total volume of 25 μl and included the following components: cDNA derived from 25 ng of total RNA, 400 nM of each primer, RNase-free water, and 12.5 μl of SYBR Green PCR Master Mix (ABI), an optimised buffer system containing AmpliTaq Gold DNA polymerase and dNTPs. All PCR reactions were performed in duplicate and cycling parameters were as follows: after an initial denaturation step for 10 min at 95°C, 40 subsequent cycles were performed in which samples were denatured for 15 s at 95°C followed by primer annealing and elongation at 60°C for 1 min. The relative quantities of mRNA were normalised by 18S rRNA content.

### Statistical analyses

**Fig 2:**
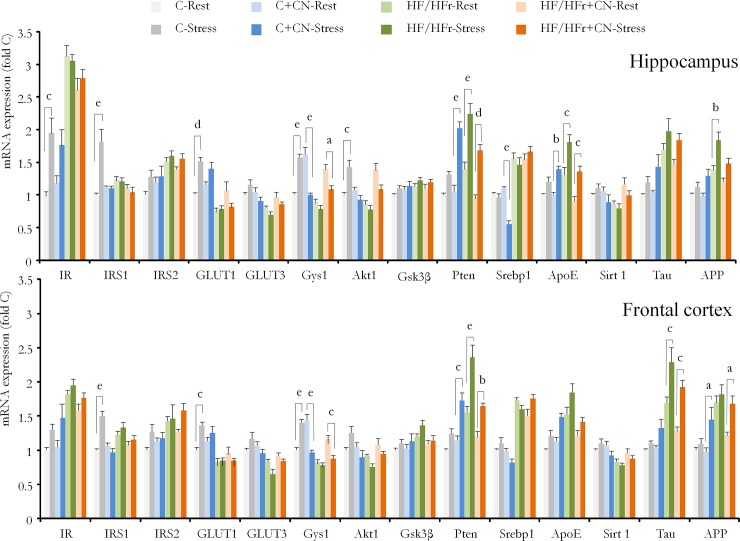
The expression of mRNA in the hippocampus and frontal cortex. The rats were fed with control (C) or high fat/high fructose (HF/HFr) diets supplemented or not with cinnamon (CN) for 12 weeks, then exposed (Stress) or not (Rest) before the sacrifice. The 8 groups were the following: C (Rest: n = 8, Stress: n = 8), HF/HFr (Rest: n = 8, Stress: n = 8), C+CN (Rest: n = 9, Stress: n = 7) and HF/HFr+CN (Rest: n = 8, Stress: n = 8). Comparisons are made using three-way ANOVA followed by Bonferroni *post-hoc* tests for all couples. Here are reported only those concerning the Rest *vs*. Stress conditions in each diet. Results are expressed as following: a: p<0.10, b: p<0.05, c: p<0.01, d: p<0.001 and e: p<0.0001. Values are expressed as mean ± SEM.

The statistical analyses were carried out using Statistica 7.1 software (Statsoft France, Maison-Alford). The effects of stress on C, HF/HFr, C+CN and HF/HFr+CN fed rats were evaluated using a three-may ANOVA (Stress, Diet, Cinnamon) followed, if necessary, by *post-hoc* Bonferroni tests for all couples. The impact of stress on C+CN, HF/HFr, HF/HFr+CN was studied using the interactions Stress x CN, Stress x HF/HFr and Stress x HF/HFr+CN, respectively. The p value results are presented in Table 1. The effective stress-induced differences were evaluated for each mRNA gene expression using Bonferroni *post-hoc* test comparing stressed *vs*. unstressed animals in each diet. The results are reported in Fig 2. The specific effects of each diet in the absence of stress are reported elsewhere [5].

**Table 1:** p-value results of the three-way ANOVA carried out on hippocampus and frontal cortex mRNA expressions.

	*Ir*	*Irs1*	*Irs2*	*Glut1*	*Glut3*	*Gys1*	*Gsk3*	*Akt1*	*Pten*	*Srebp1*	*ApoE*	*Tau*	*App*	*Sirt1*
**Hippocampus**														
**Diet**	<0.0001	ns	<0.0001	<0.0001	<0.0001	<0.0001	<0.05	ns	<0.01	<0.0001	<0.001	<0.0001	<0.0001	ns
**CN**	= 0.0878	<0.001	ns	= 0.0606	ns	<0.001	ns	= 0.0775	ns	ns	<0.01	ns	ns	= 0.0797
**Stress**	<0.001	<0.01	<0.05	<0.05	ns	<0.05	<0.05	ns	<0.0001	<0.01	<0.0001	<0.001	<0.0001	ns
**Diet x CN**	= 0.0952	ns	ns	ns	<0.01	<0.001	ns	<0.0001	<0.0001	<0.05	<0.0001	= 0.0642	<0.01	<0.01
**Stress x Diet**	<0.01	<0.001	ns	<0.0001	ns	= 0.0816	ns	<0.01	ns	<0.01	ns	ns	ns	ns
**Stress x CN**	ns	<0.01	ns	<0.05	= 0.0762	<0.0001	ns	<0.001	<0.05	ns	ns	ns	ns	= 0.0782
**Stress x Diet x CN**	ns	<0.01	ns	ns	= 0.0812	<0.0001	ns	ns	<0.01	<0.01	ns	ns	ns	ns
**Frontal cortex**														
**Diet**	<0.0001	ns	<0.001	<0.0001	<0.0001	<0.0001	<0.01	<0.05	<0.0001	<0.0001	<0.0001	<0.0001	<0.0001	<0.001
**CN**	ns	<0.0001	ns	ns	ns	<0.01	ns	ns	ns	= 0.0535	ns	= 0.0818	ns	ns
**Stress**	<0.001	<0.01	<0.05	<0.05	ns	<0.01	<0.05	ns	<0.0001	ns	<0.0001	<0.0001	<0.001	ns
**Diet x CN**	<0.05	ns	ns	ns	<0.05	<0.01	= 0.0510	<0.01	<0.0001	ns	<0.0001	<0.001	<0.01	<0.05
**Stress x Diet**	ns	ns	ns	<0.01	= 0.0721	= 0.0799	ns	<0.05	ns	ns	ns	<0.01	ns	ns
**Stress x CN**	ns	<0.001	ns	<0.05	ns	<0.0001	ns	= 0.0646	ns	ns	ns	ns	<0.05	= 0.0667
**Stress x Diet x CN**	ns	<0.01	ns	ns	<0.05	<0.0001	ns	<0.05	<0.05	<0.001	ns	ns	ns	ns

The rats were fed for 12 weeks with control (C), high fat/high fructose (HF/HFr) diets with a supplement of cinnamon (CN) or not, then exposed (Stress) or not (Rest) to a 30-min restraint+10-min open-field before the sacrifice. There were 8 groups: C (Rest: n = 8, Stress: n = 8), HF/HFr (Rest: n = 8, Stress: n = 8), C+CN (Rest: n = 9, Stress: n = 7) and HF/HFr+CN (Rest: n = 8, Stress: n = 8). Comparisons are made using three-way factorial ANOVA with Stress, HF/HFr (Diet) and cinnamon (CN) effects and Interactions. The p values are given for ANOVA effects and interactions. The threshold for statistical signification was set at p<0.05 and trends were considered at p<0.10.

The relations among all the mRNA expressions were evaluated in C rats using factorial analysis with normalised Varimax to calculate the weight of each component for each factor. Factors were considered if their Eigenvalue exceeded 1. Components of the factors were considered if their weight exceeded 0.70. For this analysis, the hippocampus (h) and frontal cortex (fc) mRNA were assembled as they were submitted to the same history of diet and stress.

The relations between blood corticosterone and changes in brain mRNA expression were studied in each diet using multiple regression tests with corticosterone as the predictor. Resting (n = 8) and stressed (n = 8) animals were combined to take into account the high dynamic range of biological response. However, the analyses were done separately in the hippocampus and frontal cortex to respect their differences in glucocorticoid effects. To be conservative, only strongly adjusted multiple correlations (r^2^>0.40) were taken into account.

For all analyses, the threshold for statistical significance was set at 0.05. However, trends were considered at p<0.10. The values are given as mean ± SEM.

## Results

### Behavioural results

All stressed animals exhibited the same defecation level during the acute stress exposure and the same behaviour in the open field (Table 2, S2 Table).

**Table 2:** Behaviour expressed by rats in open field immediately after a 30-min restraint.

	C		HF/HFr	C+CN	HF/HFr+CN
**Defecation**	3.4±0.6		5.2±0.9	3.6±0.6	5.3±0.9
**Entire open field**					
**Grooming Nb**		2.8±0.5	2.6±0.8	2.9±0.7	1.6±0.2
**Grooming duration**		60±8	58±13	58±15	29±5
**Rearing Nb**		22.6±3.8	18.2±3.4	21.6±2.2	18.8±3.8
**Rearing duration**		41±6	37±8	45±6	38±8
**Inactivity duration**		465±25	490±17	464±21	489±22
**Distance**		3329±618	2690±330	3140±356	2668±396
**Center of the open field**					
**Entries Nb**		6.5±2.6	6.8±2.0	5.4±1.5	5.0±1.1
**Inactivity duration**		14±4	9±4	8±3	12±6
**Duration in the center**		34±8	26±7	28±8	25±9
**Distance**		461±177	363±85	375±125	268±58

The rats were fed with control (C, n = 10), high fat/high fructose (HF/HFr, n = 10), control+cinnamon (C+CN, n = 10), and high fat/high fructose+cinnamon (HF/HFr+CN, n = 10) diets for 12 weeks. Distance is given in cm and duration in s. Values are expressed as mean ± SEM.

### Stress-induced changes in mRNA expression in control animals

The factorial analysis (h: hippocampus and fc: frontal cortex) isolated 5 factors that explained 87% of the global variance. The factors (variables, percentage of explained variance) were the following: factor 1 (h-*Irs1*, fc-*Irs1*, h-*Pten*, fc-*Pten*, h-*ApoE*, fc-*ApoE*, fc-*Sbrep1*; 41%), factor 2 (h-*Ir*, h-*Akt*, fc-*Akt*, h-*Gsk3*, fc-*Gsk3*; 19%), factor 3 (h-*Glut3*, fc-*Glut3*, h-*App*, h-*Sirt1*, fc-*Sirt1*; 15%), factor 4 (h-*Irs2*, fc-*Irs2*; 7%) and factor 5 (no component weighing more than 0.70; 4%). Hippocampus and frontal cortex gene expressions were coordinated as both areas were found for the same gene in the same factor. The sole discordance are fc-*Ir* in the factor 2 (weight: 0.6823) and fc-*App* in the factor 3 (weight: 0.6913), both being near the threshold.

In the hippocampus (Table 1, S1 Table), stress induced a clear increase in mRNA gene expression of *Ir* (p<0.001), *Irs1* (p<0.01), *Ir2* (p<0.05), *Glut1* (p<0.05), *Gys1* (p<0.05), *Gsk3* (p<0.05), *Pten* (p<0.0001), *ApoE* (p<0.0001), *Tau* (p<0.001) and *App* (p<0.0001), but a decrease in *Sbrep1* (p<0.01). In the frontal cortex (Table 1, S1 Table), stressor exposure was followed by an increase in mRNA gene expression of *Ir* (p<0.001), *Irs1* (p<0.01), *Ir2* (p<0.05), *Glut1* (p<0.05), *Gys1* (p<0.01), *Gsk3* (p<0.05), *Pten* (p<0.0001), *ApoE* (p<0.0001), *Tau* (p<0.0001) and *App* (p<0.001).

In Control animals (Fig 2), stressor exposure resulted in an increase in *Ir* (p<0.01), *Irs1* (p<0.0001), *Glut1* (p<0.001), *Gys1* (p<0.0001) and *Akt1* (p<0.01) in the hippocampus. In the frontal cortex (Fig 2), the increase in mRNA gene expression is observed for *Irs1* (p<0.0001), *Glut1* (0.01) and *Gys1* (p<0.0001).

### Stress-induced changes in mRNA expression in C+CN rats

Animals supplemented with CN did not exhibit the same stress-induced mRNA gene expression than rats without any supplement. In the hippocampus (Table 1), the interaction between Stress and CN effects was significant for *Irs1* (p<0.01), *Glut1* (p<0.05), *Glut3* (p<0.10), *Gys1* (p<0.0001), *Akt1* (p<0.001), *Pten* (p<0.05), and *Sirt1* (p<0.10). In the frontal cortex (Table 1), the interaction was observed for *Irs1* (p<0.01), *Glut1* (p<0.05), *Gys1* (p<0.0001), *Akt1* (p<0.10), *App* (p<0.05) and *Sirt1* (p<0.10). It resulted in an increase in hippocampus mRNA gene expression of *Pten* (p<0.0001), *ApoE* (p<0.05) and a decrease in *Gys1* (p<0.0001) and *Sbrep1* (p<0.0001) in Stress C+CN rats compared to Rest C+CN rats (Fig 2),. In the frontal cortex (Fig 2), the stress-induced increase concerned *Pten* (p<0.01) and *App* (p<0.10) while a decrease was observed in *Gys1* (p<0.0001) (Fig 2).

### Stress-induced changes in mRNA expression in HF/HFr rats

When HF/HFr fed animals were exposed to stress, a clear interaction between their diet and the effect of stress was observed (Table 1). In the hippocampus, the interaction between Diet and Stress concerned the mRNA gene expression of *Ir* (p<0.01), *Irs1* (p<0.001), *Glut1* (p<0.0001), *Gys1* (p<0.10), *Akt1* (p<0.01) and *Srebp1* (p<0.01). In the frontal cortex (Table 1), the interaction was observed in *Glut1* (0.01), *Glut3* (p<0.10), *Gys1* (p<0.10), *Akt1* (p<0.05) and *Tau* (p<0.01). In the hippocampus (Fig 2), there was an increase in *Pten* (p<0.0001), *ApoE* (p<0.01) and *App* (p<0.05) in Stress HF/HFr rats as compared to Rest HF/HFr rats. In the frontal cortex (Fig 2), Stress HF/HFr rats exhibited an increase in *Pten* (p<0.0001) and Tau (p<0.01) compared to Rest HF/HFr rats.

### Stress-induced changes in mRNA expression in HF/HFr+CN rats

The HF/HFr+CN diet also altered the stress-induced mRNA expression as suggested by the interaction between stress and diet and cinnamon supplement (Table 1). In the hippocampus, an interaction was observed for *Irs1* (p<0.01), *Glut3* (p<0.10), *Gys1* (p<0.0001), *Pten* (p<0.01) and *Srebp1* (p<0.01). In the frontal cortex, the HF/HFr+CN diet modified the stress induced mRNA expression for *Irs1* (p<0.01), *Glut3* (p<0.05), *Gys1* (p<0.0001), *Akt1* (p<0.05), *Pten* (p<0.05) and *Srebp1* (p<0.001). Finally (Fig 2), compared to non stressed HF/HFr+CN rats, the stressed HF/HFr+CN rats exhibited higher hippocampus mRNA levels of *Pten* (p<0.001) and *ApoE* (p<0.01) but lower levels of *Gys1* mRNA (p<0.10). In the frontal cortex (Fig 2), Stress HF/HFr+CN rats exhibited higher mRNA levels of *Pten* (p<0.05), *Tau* (p<0.01) and *App* (p<0.10) but lower levels of *Gys1* (p<0.01) mRNA than Rest HF/HFr+CN rats.

### Correlation between blood corticosterone level and brain mRNA expression

In C rats, stress exposure was followed by a large increase in corticosterone (Stress, p<0.001). This effect was blunted by prior HF/HFr diet consumption (Interaction: p<0.05; Stress, p<0.001). The previous cinnamon supplement did not modify the stress-induced increase in corticosterone (C *vs*. C+CN: Stress, p<0.001 and C *vs*. HF/HFr+CN: Stress, p<0.001) (Table 3, S3 Table).

**Table 3:** Plasma corticosterone values (ng/ml) assessed on 8 experimental groups.

	Rest	Stress
**C**	59.10±12.35 (20)	555.37±72.14 (10) ***
**HF/HFr**	41.57±16.36 (20)	401.57±34.33 (10) *** #
**C+CN**	44.68±12.38 (20)	435.81±29.19 (10) ***
**HF/HFr+CN**	33.07±7.02 (20)	417.07±32.70 (9) ***

The group sizes are indicated in brackets. Comparisons were made using the two-way factorial ANOVA (diet, stress). The results are given as: Stress effect

***: p<0.001 and Interaction

#: p<0.05. Values are given as mean ± SEM.

In the C group, positive correlations were observed between blood corticosterone level and *Irs2* (hippocampus: r^2^ = 0.420, p<0.01), *Glut1* (hippocampus: r^2^ = 0.733, p<0.001; frontal cortex: r^2^ = 0.593, p<0.001), *Glut3* (hippocampus: r^2^ = 0.455, p<0.01; frontal cortex: r^2^ = 0.478, p<0.01), *Gys1* (hippocampus: r^2^ = 0.607, p<0.001), *Pten* (hippocampus: r^2^ = 0.467, p<0.01) and *Sirt1* (hippocampus: r^2^ = 0.424, p<0.01) mRNA expression levels. In the C+CN group, the sole correlation observed was between corticosterone and *Glut1* (hippocampus: r^2^ = 0.538, p<0.001). No correlation was observed within the HF/HFr and HF/HFr+CN groups.

## Discussion

The main finding of the study is that a single moderate stressor exposure was followed by an enhanced mRNA expression of genes involved in insulin signalling and cell glucose control. This stress-induced mRNA expression was blunted by the previous HF/HFr diet, CN supplement or a combination of both. However, no difference in behaviour was observed among acutely stressed rats according diets.

### The effect of stress on mRNA expression of genes involved in insulin signalling

Restraint is considered as a moderate stressor that activates the brain [42]. It induced changes in brain mRNA expression of genes involved in energy management. These modifications occurred in less than one hour. This time course is similar to that of early genes [49] and is in accordance with studies showing that *Glut1* transcription behaves like early genes [47]. The hippocampus and frontal cortex, 2 brain areas known for their role in emotion regulation, expressed *Ir* [50] and *Glut1* [51] proteins and have similar stress-induced modification in mRNA expression (our study). The concomitant increase in *Gys1* [48, 52] and *Glut1* [51] under stress-induced brain activation suggests a participation of astrocytes [53, 54]. We also observed the lack of increase in *Glut3* mRNA expression reported under stress [55]. Lastly, the stress-induced mRNA expression we observed is likely to be coordinated as the factor analysis isolates 4 factors with a biological consistence: factor 1 is related to the transduction of insulin signals, factor 2 and 3 are related to insulin signalling linked to oxidative signalling control [56] and factor 4 is related to nutrient homeostasis [57].

The value of the biological value of the stress-induced reaction is likely to be adaptive. The mRNA expression can be considered as the early phase of a coordinated diachronic reaction triggered by stress and aimed at facilitating the glucose entrance in the cerebral tissue and refilling brain with energy after the large expenditure driven by stress [58]. Although the increase in *Irs1* mRNA expression can reflect either a decreased [59], or enhanced [60, 61] insulin sensitivity, the latter is more probable as the *Akt1* mRNA expression also increased [61–63]. The increase in *Ir* concomitantly to *Glut1*, at least in the hippocampus, is in agreement with this interpretation. The increased insulin sensitivity favours therefore large brain glucose entry [58]. In line with this hypothesis, a similar reaction is triggered by other stressors: (i) a pulse of catecholamines trigger *in vitro* spontaneous synthesis of glycogen 9h later [64] and (ii) hypoglycemia is followed by an over refilling of brain glycogen [52].

### The effect of a previous HF/HFr diet on stress-induced mRNA expression

This 12-week HF/HFr diet was shown to induce peripheral [1] and brain [5] insulin resistance. The stress-induced increase in *Irs1*, *Glut1* and *Gys1* was blunted in animals fed with HF/HFr diet. This effect suggests an impairment of the stress-induced adaptive reaction. This is supported by the contrast of *Akt1* and *Pten* mRNA expression between C and HF/HFr rats ([C: ↗ *Akt1*-→ *Pten*] *vs*. [HF/HFr:→*Akt1*-↗*Pten*]) [65]. This effect observed after single exposure to a moderate stressor remained restricted to the biological level and did not reach a behavioural level as no difference in behaviour was observed in the open field test carried out shortly after stressor exposure.

Furthermore, the increase in *Akt1* mRNA expression in C group supports a neuroprotective effect as the insulin is neuroprotective through the *Akt1* and *Gsk3β* pathway [56, 66], the Akt target being generic for neuroprotection [67]. Conversely, the blockade of the stress-induced increase in *Akt1* mRNA expression in HF/HFr group suggested a decrease in a neuroprotective effect.

### The effect of a previous CN supplement on the stress-induced mRNA expression

The cinnamon supplement also modified the changes observed in mRNA expression after stress exposure. The stress-induced enhancement in *Glut1* mRNA expression observed in C rats was limited after CN supplement. However, the stress-induced increase in *Gys1* mRNA expression observed in C animals was not seen in C+CN animals that exhibited rather a decrease in *Gys1* expression as compared to their resting condition. The high *Gys1* expression observed in Rest C+CN animals is congruent to the increase in glycogen observed in the liver of resting animals after the CN supplement [68]. All that suggests that the stress-induced increase in need of glucose was less important in C+CN rats.

The CN supplement has been shown to produce a neuroprotective effect [34]. In our case, the pattern of stress-induced change in *Akt1*, *Gsk3β* and *Pten* was similar in C+CN and HF/HFr diets. The lack of Akt1 increase associated with the Pten increase favours a deleterious effect [65]. However, the HF/HFr rats differed from the C+CN rats by their *Srebp1* mRNA expression. *Srebp1* participates in fatty acid biosynthesis regulation [69] and favours membrane fluidity [70]. A decrease in *Srebp1* after stress in C+CN rats suggests that there is no assistance in maintaining membrane fluidity, a situation of low allostatic load [70]. The specific decrease in *Srebp1* mRNA expression after stress indicates a neuroprotective effect. Accordingly, brain *Srebp1* mRNA expression is increased after a high fat diet [5] and aging [71], both situations related to a high allostatic load.

### Role of glucocorticoid on stress-induced mRNA expression

During this early stress phase, the high concentration of glucocorticoids may have an important modulator effect on the brain, especially on the insulin pathway [72]. In C rats, blood corticosterone levels were positively correlated with *Irs2*, *Glut1*, *Glut3*, *Gys1*, *Pten* and *Sirt1*. Thus suggests that glucocorticoid favours glucose use by neurons and astrocytes, either immediately or directly afterwards. This effect was only partly observed in C+CN rats in accordance with the effect of cinnamon on the stress-induced changes in mRNA expression. However, this effect of glucocorticoids was blunted in HF/HFr and HF/HFr+CN rats, suggesting that their brain energy regulation becomes partly independent from blood glucocorticoid levels.

### Conclusion

In conclusion, a single exposure to a moderate stressor is followed by genomic activation favouring replenishment of cell energy, an effect congruent to an adaptive response. This suggests that a moderate stress favours energy refilling mechanisms, an effect blunted by a previous HF/HFr diet and cinnamon supplement.

## Supporting information

S1 Table Data related to molecular biology analysis. (XLSX)

S2 Table Data related to behavioral test. (XLSX)

S3 Table Data related to plasma corticosterone values. (XLSX)
